# Failure Mechanism and Control Mechanism of Intermittent Jointed Rock Bridge Based on Acoustic Emission (AE) and Digital Image Correlation (DIC)

**DOI:** 10.3390/ma17133190

**Published:** 2024-06-29

**Authors:** Hang Lin, Xing Zhang, Su Li

**Affiliations:** School of Resources and Safety Engineering, Central South University, Changsha 410083, China or hanglin@csu.edu.cn (H.L.); zhangxing1994@csu.edu.cn (X.Z.)

**Keywords:** deep excavation, intermittent joint, DIC technology, AE technology, failure mode

## Abstract

Deep foundation pit excavation is an important way to develop underground space in congested urban areas. Rock bridges prevent the interconnection of joints and control the deformation and failure of the rock mass caused by excavation for foundation pits. However, few studies have considered the acoustic properties and strain field evolution of rock bridges. To investigate the control mechanisms of rock bridges in intermittent joints, jointed specimens with varying rock bridge length and angle were prepared and subjected to laboratory uniaxial compression tests, employing acoustic emission (AE) and digital image correlation (DIC) techniques. The results indicated a linear and positive correlation between uniaxial compressive strength and length, and a non-linear and negative correlation with angle. Moreover, AE counts and cumulative AE counts increased with loading, suggesting surges due to the propagation and coalescence of wing and macroscopic cracks. Analysis of RA-AF values revealed that shear microcracks dominated the failure, with the ratio of shear microcracks increasing as length decreased and angle increased. Notably, angle exerted a more significant impact on the damage form. As length diminished, the failure plane’s transition across the rock bridge shifted from a complex coalescence of shear cracks to a direct merger of only coplanar shear cracks, reducing the number of tensile cracks required for failure initiation. The larger the angle, the higher the degree of coalescence of the rock bridge and, consequently, the fewer tensile cracks required for failure. The decrease of length and the increase of angle make rock mass more fragile. The more inclined the failure mode is to shear failure, the smaller the damage required for failure, and the more prone the areas is to rock mass disaster. These findings can provide theoretical guidance for the deformation and control of deep foundation pits.

## 1. Introduction

The extensive and efficient utilization of underground space is an important way to ensure the sustainable development of congested urban areas. The development and utilization of all kinds of underground space involves deep foundation pit engineering. Foundation pit excavation can break the stress balance of the surrounding geotechnical masses, cause deformation of the rock mass [1,2,3,4], and present a hidden danger of instability, which threatens the safety of the surrounding residents and building facilities [5]. Pre-existing intermittent joints are the key areas that control the deformation and failure of the rock mass [6,7]. The tips of these joints are under the action of external forces, due to the stress concentration cracks continuing to sprout and expand until they intersect with adjacent cracks and joints, forming a macroscopic failure surface [8,9], which in turn affects the stability of the foundation pit engineering.

To ensure the safety and stability of excavation, in-depth studies have been conducted on the failure mechanisms of intermittent joints, yielding significant insights [10,11]. Huang et al. [12] utilized discrete element software to simulate rocky slopes with varying intermittent joint patterns and identified three distinct step failure modes: shear, tensile, and mixed tensile-shear. They also derived a formula to calculate safety coefficients under these modes, taking into account the joint aggregation degree. Liu and Dai [13] introduced a damage constitutive model that captures both the deformation and strength characteristics of a rock mass with intermittent joints under uniaxial cyclic compression. This model accounts for the coupled damage of microdefects and macro joints and successfully reproduces the hysteretic stress-strain curve and cumulative fatigue plastic deformation of rock materials under cyclic loading. Yang et al. [14] integrated the LT criterion with the Numerical Manifold Method (NMM) to ascertain the direction and length of joint extension. Zhang et al. [15] conducted laboratory experiments on two types of intermittent joints, exploring the failure mechanism of rock bridges within these joints through thermography and thermodynamic theory. Wang et al. [16] developed a limit equilibrium model combined with the fracture mechanics (FM) method to predict the stability of anti-dip bedding rock slopes with very low persistence of cross joints. To fully characterize the roughness of joints, Leal-Gomes and Dinis-da-Gama [17] put forward the formula of a strain energy limit equilibrium model of rock joints.

Estimating material properties is an essential part of any engineering field. These studies have substantially augmented our comprehension of intermittent joints. However, most research has focused on joints of varying geometries, with less consideration given to rock bridges with different geometries, particularly in conjunction with AE and DIC monitoring. Given that rock masses commonly contain numerous and disorganized intermittent joints [18,19], even two ostensibly similar joints may exhibit distinct mechanical properties due to the varied spatial distribution of the rock bridges [7,20]. It is thus imperative to investigate the controlling effects and failure modes of rock bridges within intermittent joints. This paper examines the impact of rock bridges’ length and angle on the mechanical properties of rock masses based on laboratory tests. Additionally, the AE technique is used to qualitatively and quantitatively analyze the crack evolution law. Finally, using DIC techniques, the strain field evolution and failure mode throughout the entire process are meticulously analyzed, and the mechanical properties of intermittent jointed rock mass materials are evaluated in detail, which provides a theoretical basis for predicting and controlling disasters regarding deep foundation pit rock mass.

## 2. Specimen Preparation and Test Methods

### 2.1. Specimen Preparation

**Figure 1:**
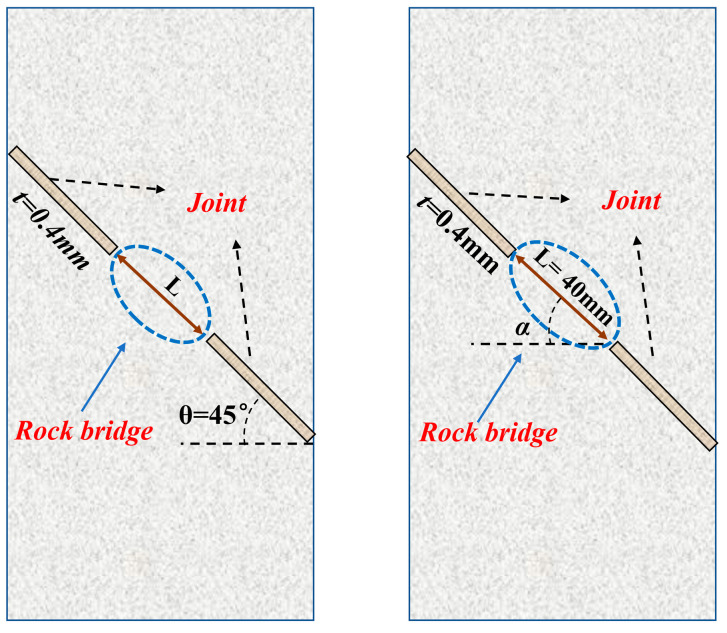
Schematic of specimens containing rock bridge with different geometric parameters.

The laboratory testing was carried out using rock-like materials prepared from cement mortar, considering that cement mortar has particles and bonding materials that are similar to the structural composition of natural rocks [21,22]. The specimen was mixed with 42.5 white silicate cement, fine sand, and water; the volume ratio was cement–fine sand–water = 2:1:1, and the specimen size was 140 mm × 70 mm × 30 mm. According to the different scales and spatial distributions of rock bridges in actual engineering, two groups of specimens were designed and fabricated by combining their common geometric distribution characteristics. The first group was the specimens with different L, of 40 mm, 35 mm, 30 mm, 25 mm, and 20 mm. The width of the joints was 0.4 mm, and joint angle was 45°. The second group was the specimens with different α, ranging from 0° to 90°. The L was fixed at 40 mm, the width of joints was 0.4 mm, and the joint angle was 45°. For each geometrical parameter of the rock bridge, two parallel specimens were fabricated to reduce experimental errors. The specimen geometry is shown in Figure 1.

**Figure 2:**
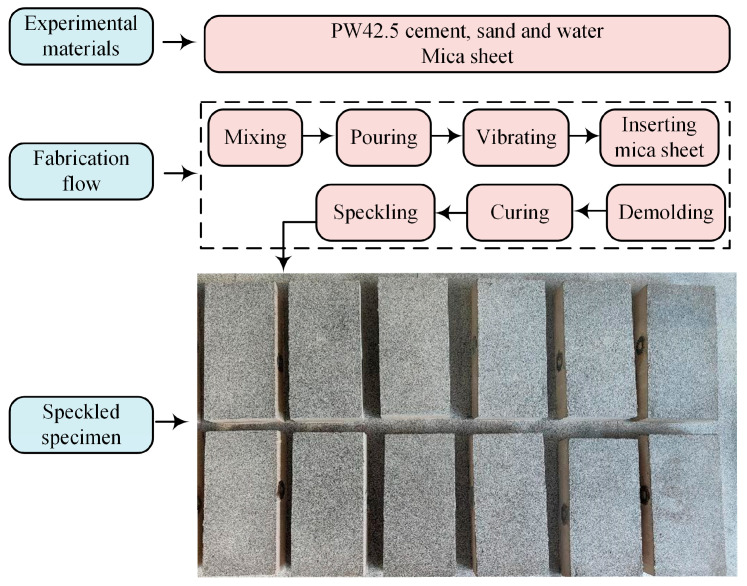
Specimen fabrication flowchart and speckled specimens.

After pouring and initial solidification, the pre-cut mica sheet was vertically inserted into the cement mortar to simulate the joint. When the final solidification was completed, the specimen was put into clean water for maintenance, and after one week, the specimen was taken out and moved to a dry and ventilated place to continue the maintenance for three weeks. Subsequent to maintenance activities, superfluous mica sheet material was meticulously trimmed from the specimen, resulting in the formation of a joint with significantly reduced strength relative to the remaining specimen structure. To facilitate the Digital Image Correlation (DIC) analysis, the specimens were then speckled. The specimen fabrication flow and speckled specimens are shown in Figure 2.

### 2.2. Test Methodology

**Figure 3:**
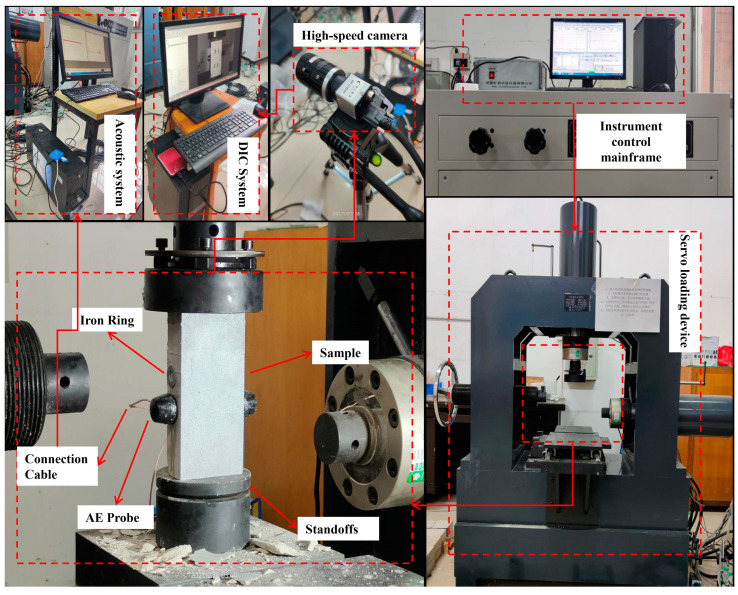
Schematic diagram of the whole test loading and monitoring system.

The main instruments and equipment used in this test are multifunctional rock shear, an acoustic emission meter, and a CCD camera. The uniaxial compression experiment was conducted on the YZW100 multifunctional shear instrument manufactured by Jinankuangyan, Jinan, China, which can provide a maximum normal force and maximum tangential force of 500 KN and has various loading modes, such as displacement control and force control. The DIC system mainly consists of a CCD camera responsible for image acquisition and the software system responsible for acquisition control and image processing analysis. The CCD camara has 5 million pixels and a resolution of 2448 × 2048, which can provide a maximum recording speed of 75 frames per second (FPS). In this research, the recording speed is set at 15 FPS. The AE meter is a PAC-II AE meter manufactured by Physical Acoustics, West Windsor Township, NJ, USA, with an amplifier frequency range of 1 khzkHz–3 mhzmHz, a sampling rate setting of 1MSPS, and a threshold setting of 40 db; the whole test system is shown in Figure 3.

The loading process is mainly divided into two stages: (1) pre-loading stage: the loading mode is force control, and the loading rate is 0.2 kN/s. The loading target is 2 kN, and it is kept for 20 s after the loading target is achieved; (2) the loading stage: the loading mode is displacement control. Given the specimen dimensions and the precision capabilities of the testing apparatus, the loading rate was established at 0.004 mm/s, with a loading target of 5.0 mm. Upon specimen failure, the loading was promptly discontinued.

## 3. Results and Discussion

### 3.1. Stress-Strain Curves

**Figure 4:**
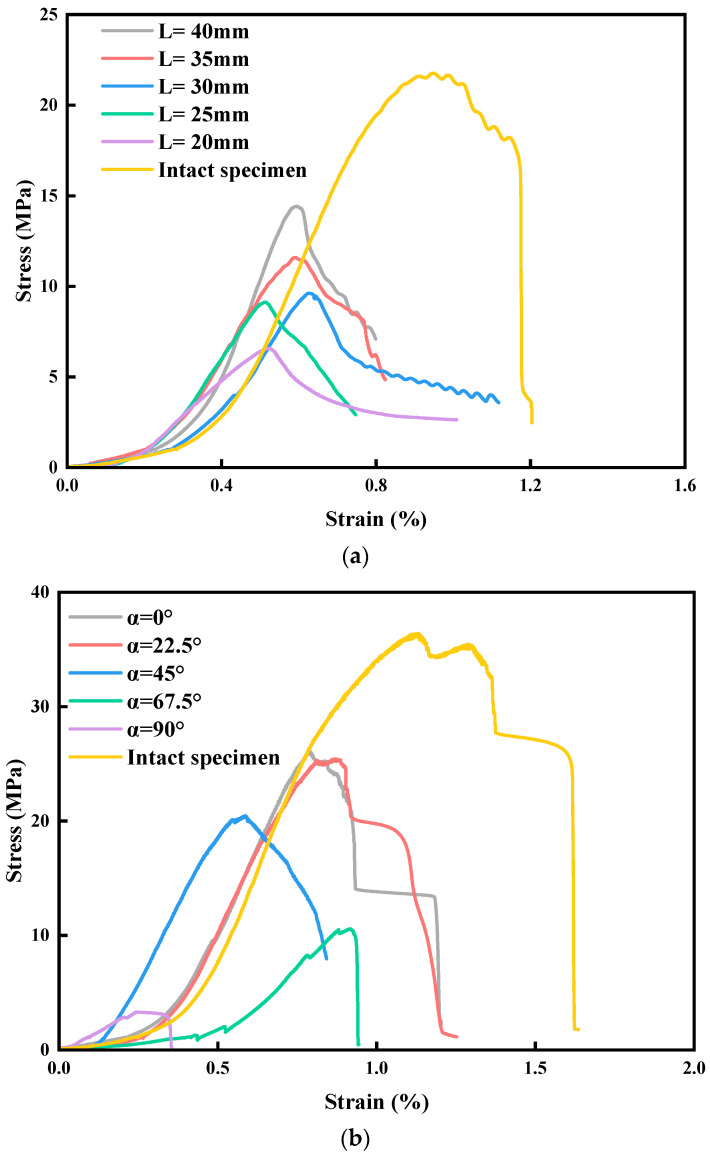
Specimens’ stress-strain curves. (**a**) Specimens with different L. (**b**) Specimens with different α.

**Figure 5:**
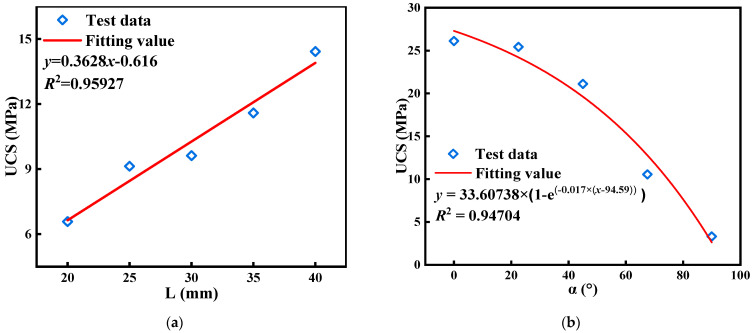
Uniaxial compressive strength versus L and α. (**a**) Specimens with different L. (**b**) Specimens with different α.

The stress-strain curves and uniaxial compressive strengths of intact specimens and jointed specimens with different L and α are shown in Figure 4 and Figure 5. The overall trends of the stress-strain curves of the specimens under uniaxial loading conditions are relatively similar and can be roughly divided into the following stages: (1) compaction stage; (2) elastic deformation stage; (3) elastoplastic deformation stage; and (4) failure (post-peak) stage. Compared with the curve of the intact specimen, the curves for jointed specimens with different L show obvious ductile characteristics, which is caused by the rock bridge of specimens in this group being coplanar with the prefabricated joints. A new continuous structural plane is formed after the rock bridge coalesces with prefabricated joints, and the upper part of specimen slides slowly along the new continuous structural plane, forming a compression-shear action until the specimen is completely destroyed. The smaller L is, the smaller the slope of the elastic stage of the curve (elastic modulus), indicating that the initial damage of the specimen is larger [23]. α also has a significant effect on the curve. When α ≤ 45°, the post-peak curve shows a stepwise decline, indicating that the specimen still has a certain pressure-bearing capacity after failure, which is because the pre-fabricated joint and the extended cracks form a new continuous structural plane after coalescence. When α > 45°, the curve in the post-peak stage decreases vertically, indicating that the specimen undergoes brittle failure, and the rock mass does not have the pressure-bearing capacity after coalescence. The larger α is, the smaller the slope of the elastic stage of the curve (elastic modulus), which characterizes a larger initial damage.

With the increase of L, the uniaxial compressive strength (UCS) of specimens increases sequentially and has a strong linear correlation, which is caused by the strong controlling effect of L on UCS. The UCS decreases nonlinearly with the increase of α. When α > 45°, the UCS of the specimen decreases more substantially. This is caused by the fact that the larger α is, the more easily the specimen becomes a failure structure.

### 3.2. Acoustic Analysis

To study the failure mechanism and mode of specimens more deeply, the variation in AE counts, b-values, and RA-AF values during the whole loading process are adopted to analyze AE characteristics and failure modes of specimens with different rock bridge geometry.

#### 3.2.1. AE Counts

**Figure 6:**
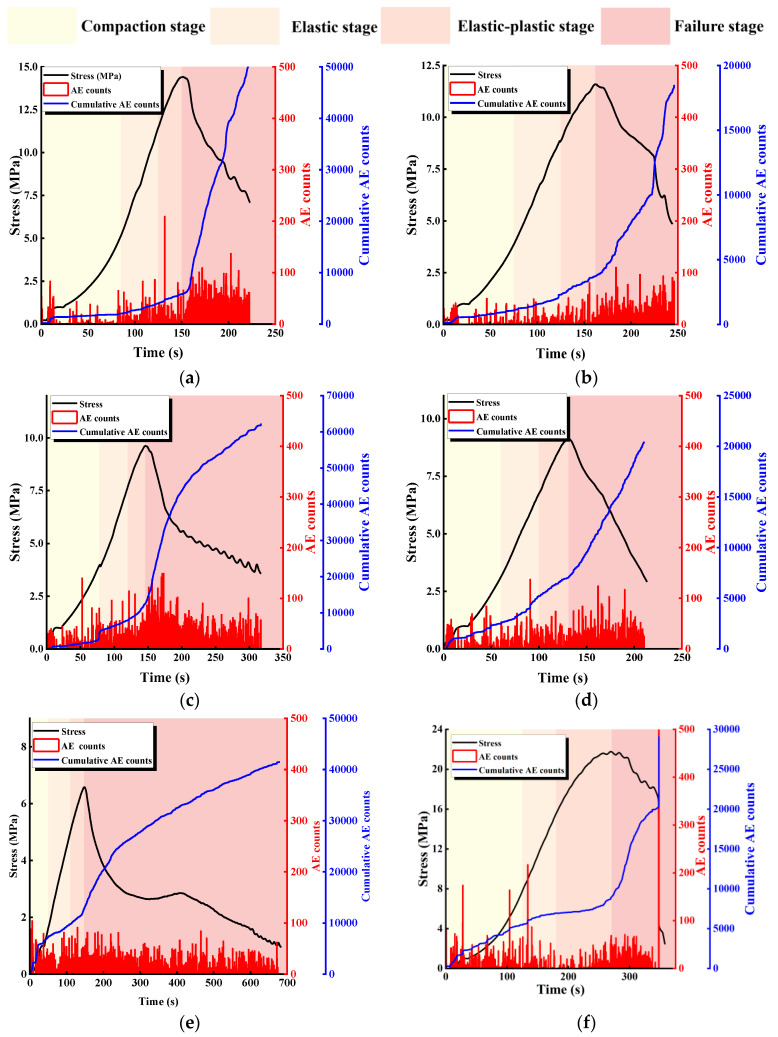
Relationship between stress and ringing count and the cumulative ringing count of specimens with different L. (**a**) L = 40 mm; (**b**) L = 35 mm; (**c**) L = 30 mm; (**d**) L = 25 mm; (**e**) L = 30 mm; (**f**) intact specimens.

Figure 6 shows the relationship between stress and ringing count and the cumulative ringing count of specimens containing different L. The characteristics of the AE signals vary within different loading stages. There is a small growth peak at the beginning of the loading, and both the stress and cumulative AE counts show nonlinear variations in this period, which is related to the loading mode of the specimens; due to the use of force control in the pre-control stage, the stress grows faster, and more acoustic emission events are generated.

In the early stage of loading, the number of AE events is high, and the cumulative AE count-time curve shows a slow growth trend, which mainly originates from the compaction and closure of the primary structure. With the increasing load, the loading enters the elastic stage, and the cumulative AE count grows steadily and its slope increases compared with that of the compression-density stage, in which the primary defects inside the specimen generally close successively, and its deformation is mainly composed of elastic deformation. After the specimen enters the elastic-plastic deformation stage, the growth trend of the cumulative AE count-time curve is intensified, and the number and size of acoustic emission signals are obviously increased, which is caused by the development and expansion of microscopic cracks, wing cracks, and so on. When loading enters the failure stage, the rock bridge ruptures, the acoustic emission signals become denser, and the cumulative AE count-time curve shows a steep increase. This is the result of the rock bridge of this group of specimens being coplanar with the prefabricated joint, which constitutes a new structural plane with the prefabricated joint after the rupture, and the upper and lower parts of the specimens slipping and failing along the new joint.

**Figure 7:**
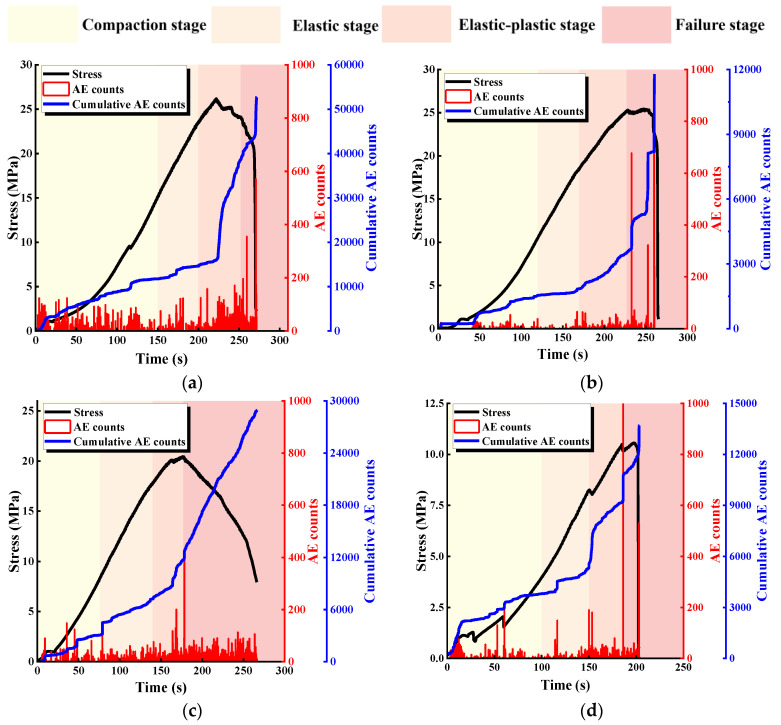

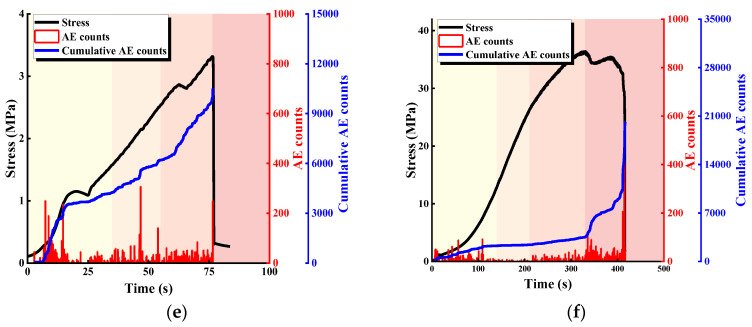
Relationship between stress and ringing count and the cumulative ringing count specimens with different α. (**a**) α = 0°; (**b**) α = 22.5°; (**c**) α = 45°; (**d**) α = 67.5°; (**e**) α = 90°; (**f**) intact specimens.

Figure 7 shows the relationship between stress and ringing count and the cumulative ringing count of specimens with different α. Despite the differences between the geometrical conditions of the rock bridges and the specimens with different L, the curves have similarities: the specimens go through the compaction stage, elastic deformation stage, elastoplastic deformation stage, and failure stage, which corresponds to compaction of the primary defects, crack initiation, extension, local penetration, and overall penetration, respectively.

Comparing the curves of specimens with different α, it can be found that when α ≤ 45°, there is an occasional slowdown before the peak of the stress-time curve, and the corresponding cumulative AE count curve has a steep increase, which is mainly caused by the expansion and development of the microscopic cracks and wing cracks. When the curve reaches the peak, the rock bridge ruptures, the specimen undergoes overall failure, and the cumulative AE count curve increases steeply accordingly. In the post-peak stage, due to the continuous slip rupture and closure of the new joint, the cumulative AE count curve then continues to rise. When α > 45°, the stress-time curves tend to slow down and drop steeply before the peak, and the corresponding cumulative AE count curve rises steeply, indicating that the expansion and development of the microscopic cracks and wing cracks are more fully developed, and the AE counts at the peak of the curve increase more compared with that of α ≤ 45°, which also indicates that the cracks at this time have a higher degree of crack development and are more fully expanded. These results indicate that as α increases, the more obvious the acoustic characteristics of the specimen, the more fully developed the specimen cracks are, and the rock mass becomes a more vulnerable structure.

#### 3.2.2. B-Values

The renowned Gutenberg-Richter (G-R) relationship equation, establishing a statistical connection between earthquake magnitude and frequency, was introduced by Gutenberg and Richter in 1941 during their comprehensive investigation of global seismic activity [24,25]:

(1) lgN=a−bM

where *M* represents the magnitude of the earthquake (amplitude of the acoustic emission signal) and *N* is the count of earthquakes with a magnitude within Δ*M*, corresponding to the number of acoustic emissions; the relationship involves constants *a* and *b*. The parameter a characterizes the seismic motion associated with the earthquake, while *b* is influenced by the distribution of acoustic emissions concerning magnitude. Notably, the b-value serves as an indicator of the scale of crack expansion.

The b-value extends beyond being a mere statistical parameter; it holds direct physical significance. It serves as an indicator of the initiation and expansion of microcracks during the process of rock failure. In the initial stages of loading, when small-scale microcracks prevail, the b-value tends to fluctuate at higher levels, and in certain rock specimens, an increase in the b-value might even be observed. However, as loading progresses, the spatial distribution of microcracks within the rock evolves from disorder to order. The prevalence of large-scale cracks increases, and clustering of acoustic emission localization events becomes more apparent. In response to these changes, the b-value undergoes a more rapid decline, reaching its lowest point when the rock destabilizes and fractures. This dynamic behavior of the b-value provides valuable insights into the evolving nature of microcrack development and the overall failure process within the rock under various loading conditions [26].

**Figure 8:**
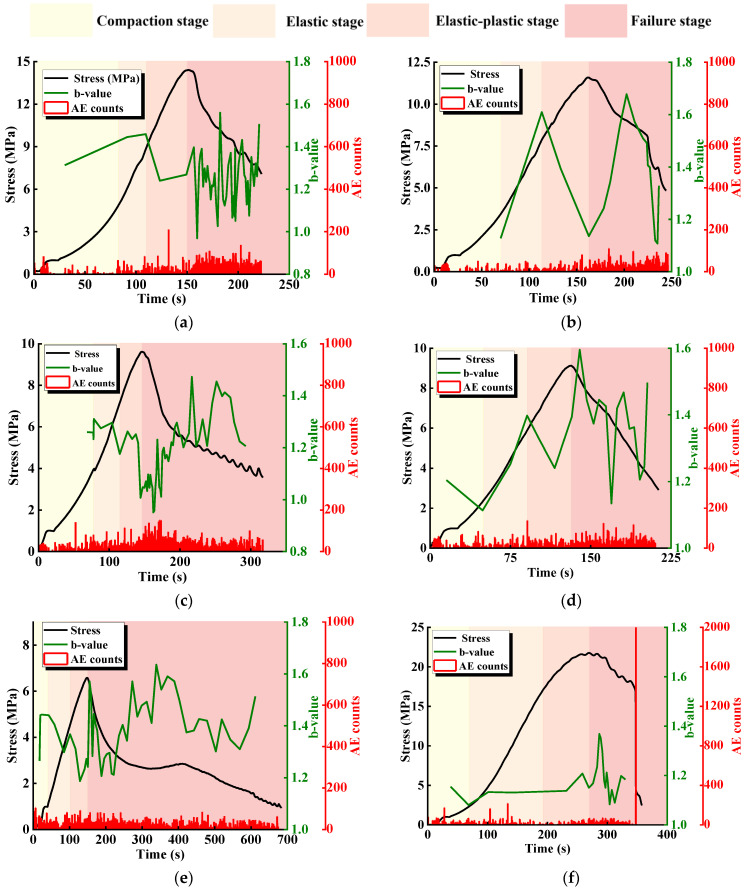
Relationship between stress and ringing count and the b-value of specimens with different L. (**a**) L = 40 mm; (**b**) L = 35 mm; (**c**) L = 30 mm; (**d**) L = 25 mm; (**e**) L = 20 mm; (**f**) intact specimens.

Figure 8 shows the relationship between stress and ringing count and the b-value of specimens with different L. B-value maxima for each specimen are about 1.5–1.7, which are larger than that for the intact specimen, which is 1.4. Based on the definition of the b-value, it can be seen that the specimens with intermittent joints are more susceptible to stress concentration at the tips of joints and other places, as well as to the further development of fissures in the course of the loading process through expansion. This series of small rupture events will contribute to the increase of b-value, while for intact specimens, fewer small rupture events will contribute to the decrease of b-value. According to the previous stress-strain characteristics and AE counting law, we know that compression loading in the compaction stage, elastic stage, and elastic-plastic stage leads to small rupture events, while large rupture events account a large proportion of the failure after the peak stage and in the specimen loading stage, as based on the law of b-values combined with the characteristics of the stress-strain curve division.

**Figure 9:**
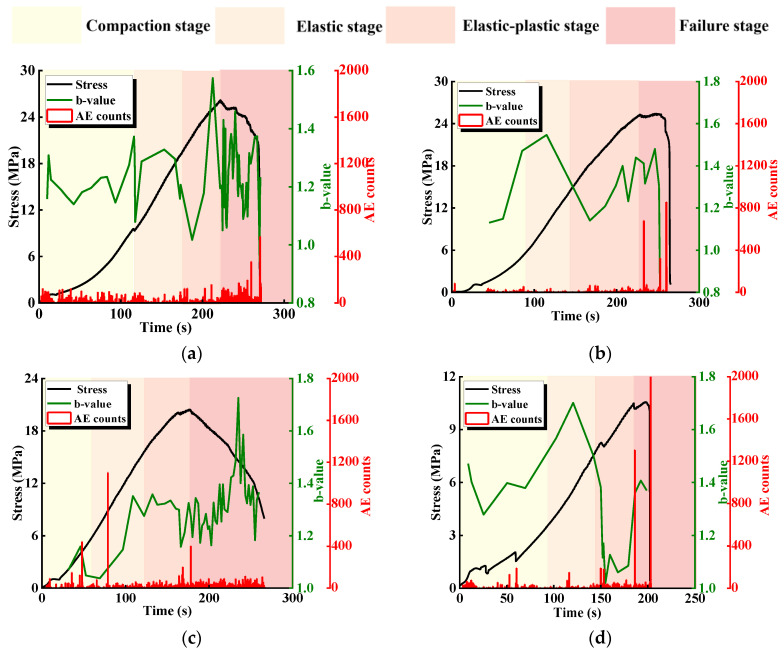

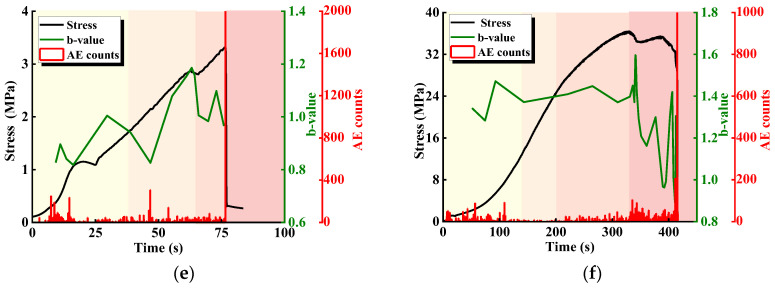
Relationship between stress and ringing count and the b-value of specimens with different α. (**a**) α = 0°; (**b**) α = 22.5°; (**c**) α = 45°; (**d**) α = 67.5°; (**e**) α = 90°; (**f**) intact specimens.

Figure 9 shows the relationship between stress and ringing count and the b-value of specimens with different α. Compared with the specimens with different L, the b-value changes in different loading stages of the specimens are similar, generally showing a rise in the compression stage and elasticity stage, with an occasional drop due to the expansion of local crack penetration or wing cracks, and a drop in the elasticity-plasticity stage, as small rupture events and large rupture events co-exist; thus, the b-value curve fluctuates. The b-value in the elastic-plastic stage is generally dominated by a decrease, and the b-value curve fluctuates due to the coexistence of small and large rupture events. In the failure stage, the new joint is constantly ruptured and closed under the pressure-shear effect, and there are more small and large rupture events; thus, the b-value curve fluctuates drastically. When α = 0° and 45°, the stress-time curve has an obvious post-peak stage, and the b-value fluctuates violently in the corresponding time period, indicating that a large number of small rupture events and large rupture events are generated in the post-peak stage, with obvious ductile characteristics. When α = 22.5° and 67.5°, the stress-time curve decreases sharply in the failure stage, and the b-value time curve decreases significantly, indicating that a large number of small rupture events and large rupture events are mainly generated in the post-peak stage, with obvious brittleness characteristics. The large number of major rupture events were mainly generated within the post-peak stage. It is worth noting that when α = 90°, due to the low strength of the specimen and the short loading process, the destruction of the specimen is dominated by macroscopic crack penetration, and the development of microscopic cracks inside the specimen is small; the maximum b-value is <1.2, which is smaller than the maximum b-value of the intact specimen, suggesting that the failure process, the mode of destruction, and the strength of the specimen have an effect on the range of the b-value as well.

#### 3.2.3. RA-AF Values

**Figure 10:**
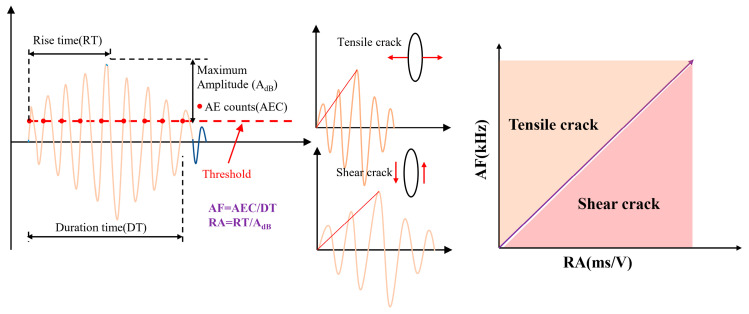
AE waveform parameters and crack classification.

RA and AF based on the acoustic emission signals are effective means of assessing the damage pattern of a rock mass. Tensile damage is transient in nature, and the acoustic emission signals generated by stretching have a short duration and rise time, so the RA value is small and the AF value is large. While shear damage is longer in duration, the duration and rise time of the acoustic emission signal generated by shear are longer, the RA value is larger, and the AF value is smaller. The equations are shown below [27]:

(2) $$\left\{ \begin{array}{l} AF=\frac{AEC}{DT}\\ RA=\frac{RT}{A_{\mathrm{db}}}\\ k=\frac{AF}{RA} \end{array}\right.$$

where *AEC* is the acoustic emission count (*AE* count), *DT* is the duration, and *AF* is in kHz. *RT* is the rise time, *A*_db_ is the amplitude, and *RA* is in ms/V. The meanings of the relevant parameters are shown in Figure 10. Since the different *RA*-*AF* characteristics of *AE* events are caused by tensile and shear microcracks, calculating the k of each *AE* event can obtain the proportion of tensile and shear microcracks during the failure process. The critical k value (the slope of the demarcation line between the tensile and shear signals) is used to distinguish tensile microcracks and shear microcracks, and the value of k for concrete samples is usually 80 [28].

**Figure 11:**
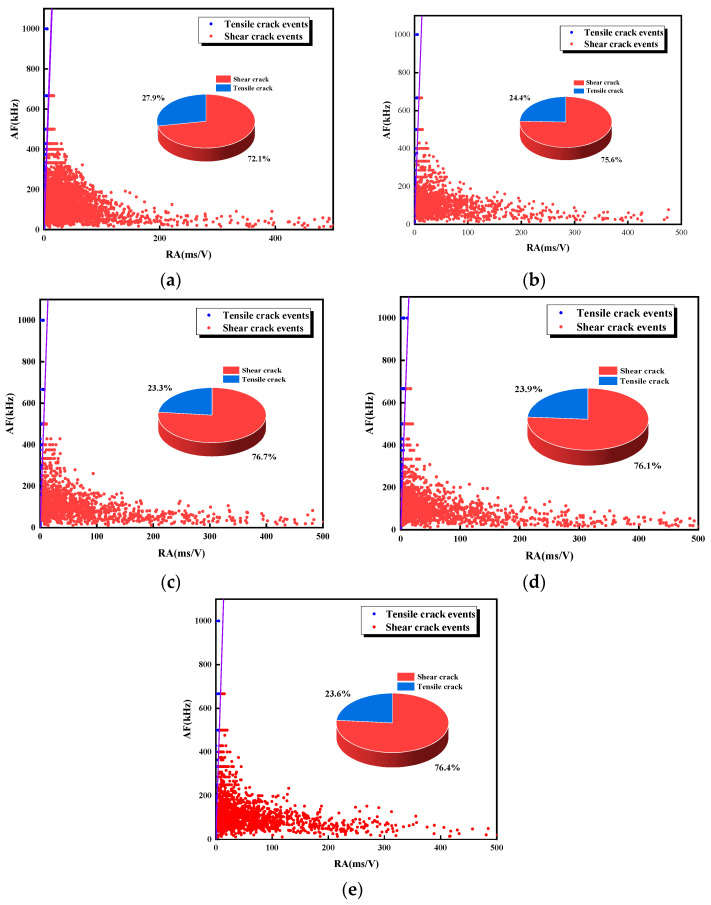
RA-AF values for specimens with different L. (**a**) L = 40 mm; (**b**) L = 35 mm; (**c**) L = 30 mm; (**d**) L = 25 mm; (**e**) L = 20 mm.

**Figure 12:**
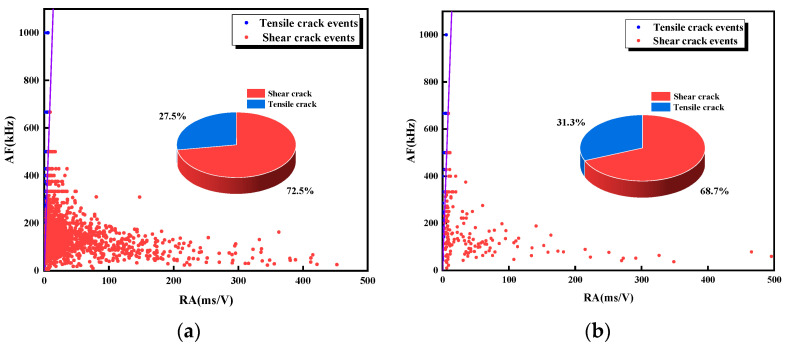

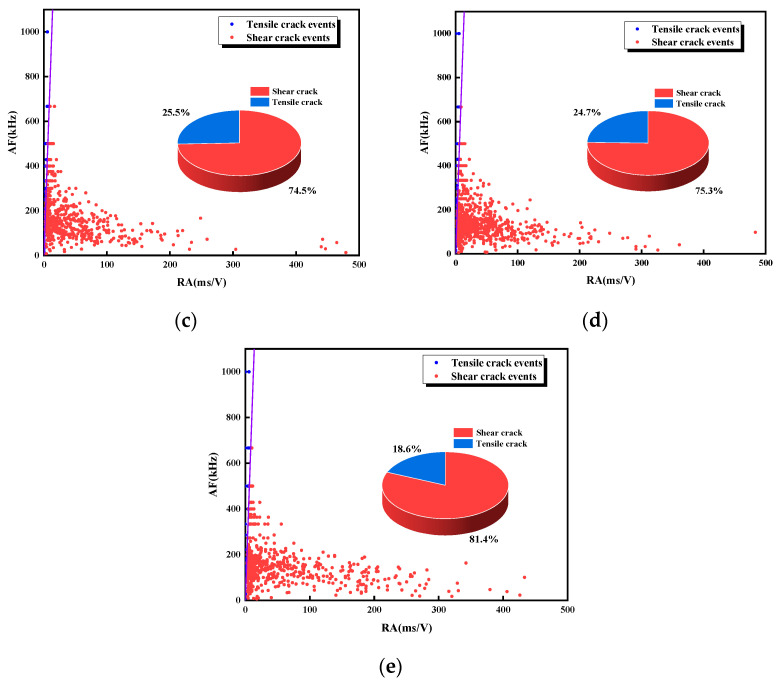
RA-AF values for specimens with different α. (**a**) α = 0°; (**b**) α = 22.5°; (**c**) α = 45°; (**d**) α = 67.5°; (**e**) α = 90°.

Figure 11 and Figure 12 show the percentage of tensile and shear signals for each specimen during loading. The number of shear signals is much larger than the number of tension signals, and shear damage is the main damage mode. As can be seen from the percentage of shear signals, the percentage of shear signals gradually increases from 72.1% to 76.4% as the L decreases. With the increase of α, the percentage of shear signal to the total signal fluctuates first and then increases, rising from 72.5% to 81.4%. It is known that when the ligament angle is small, it is difficult for coalescence to occur in the rock bridge area. The stability of the rock bridge area needs to be reduced by generating tensile cracks along the loading direction inside the rock, which will result in relatively low shear signal proportion. As the ligament angle increases, the possibility of coalescence in the rock bridge increases, and the specimen is more prone to slip along the joint direction, resulting in a higher proportion of shear microcracks. As L decreases and α increases, the shear failure degree is higher. To deeply investigate the effect of L and α on the shear signal percentage, Equation (3) is used to dimensionlessly process L and α [29]. The expressions of L and α as shown in Equation (4) are obtained. The derivation of the expressions shows that the influence coefficient of the α group is larger than that of the L group, indicating that α has a greater influence on the damage form.

(3) $$\left\{ \begin{array}{l} L^{\prime }=\frac{L-L_{\min}}{L_{\max}-L_{\min}}\\ \alpha^{\prime }=\frac{\alpha -\alpha_{\min}}{\alpha_{\max}-\alpha_{\min}} \end{array}\right.$$

where $L^{\prime }$ and $\alpha^{\prime }$ are dimensionless parameters, $L_{\max}$ and $\alpha_{\max}$ are maximum values (40 mm and 90°), and $L_{\min}$ and $\alpha_{\min}$ are minimum values (20 mm and 0°).

(4) $$\left\{ \begin{array}{l} R_{SS}=-0.003e^{\frac{L^{\prime }}{0.137}}+76.4\%\\ R_{SS}=0.519e^{\frac{\alpha^{\prime }}{0.328}}+70.4\% \end{array}\right.$$

where $R_{SS}$ is the shear signal percentage.

**Figure 13:**
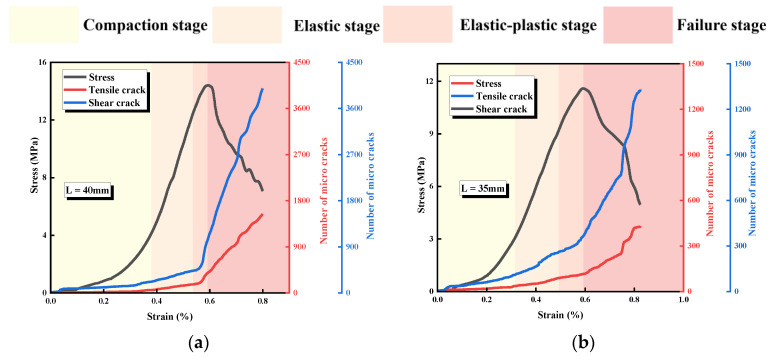

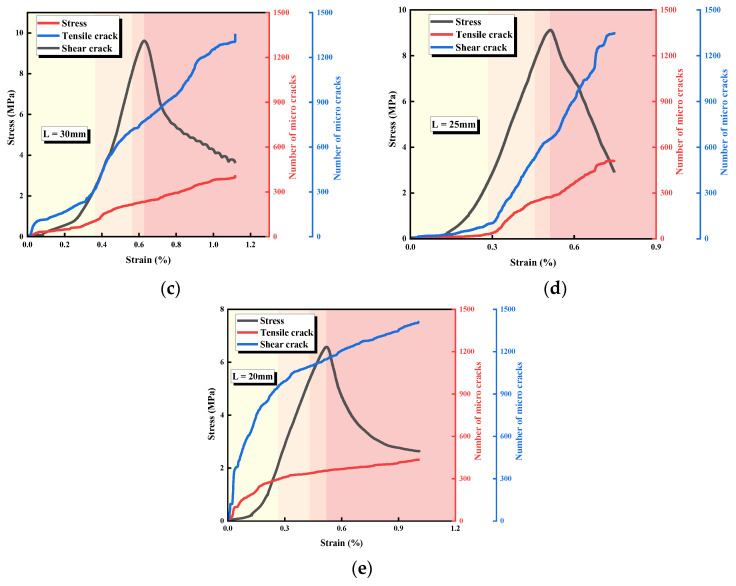
Tensile and shear microcracking evolution law for specimens with different L. (**a**) L = 40 mm; (**b**) L = 35 mm; (**c**) L = 30 mm; (**d**) L = 25 mm; (**e**) L = 20 mm.

**Figure 14:**
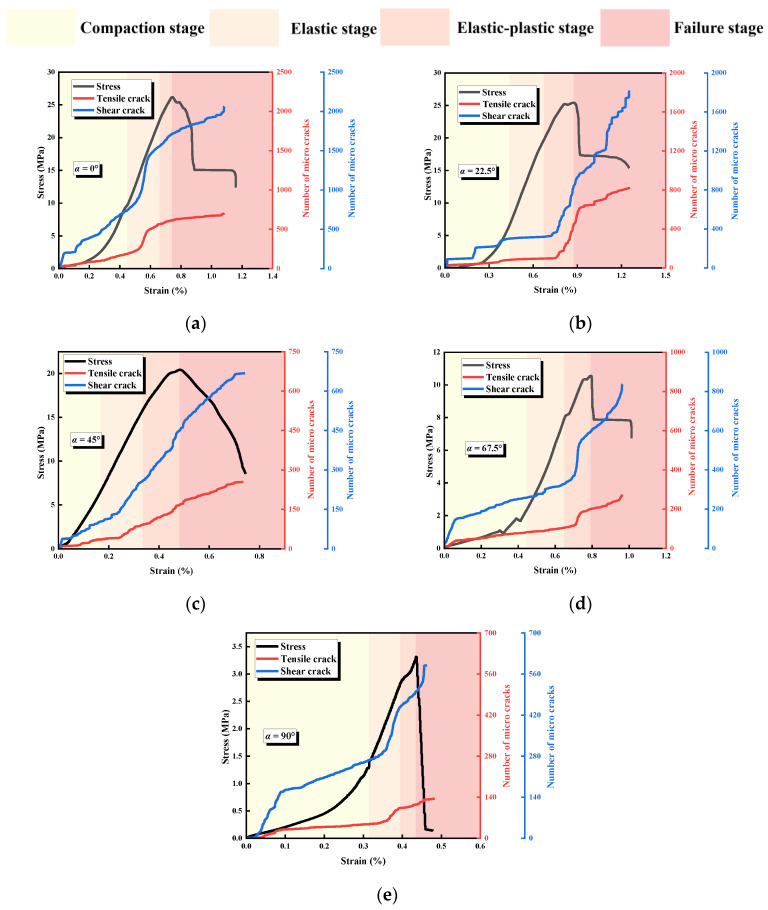
Tensile and shear microcrack evolution law for specimens with different α. (**a**) α = 0°; (**b**) α = 22.5°; (**c**) α = 45°; (**d**) α = 67.5°; (**e**) α = 90°.

To further investigate the evolution of tensile and shear microcracks during the failure process of the specimens, time was taken as the horizontal axis, the type of microcracks at each point was determined according to the relative magnitude of the RA and AF values, and the number of microcracks was recorded as 1. The number of tensile and shear microcracks was summated to obtain the evolution curves of microcracks during the failure process, which are shown in Figure 13 and Figure 14. During the uniaxial compression of the specimen, both tensile and shear microcracks increased slowly and then rapidly, and a large number of microcracks was continuously generated after the stress reached the peak. During the loading process, the number of shear microcracks in the specimen was larger than the number of tensile cracks. As the loading continued, the dominance of shear microcracks gradually increased, and the degree of shear microcrack development reached the peak at the end of loading. This indicates that shear microcracks dominate the cracks generated during the whole loading process, and this phenomenon becomes more and more obvious as the loading proceeds; the failure mode of the specimens is characterized by shear damage.

### 3.3. Failure Analysis Based on DIC Technology

**Figure 15:**
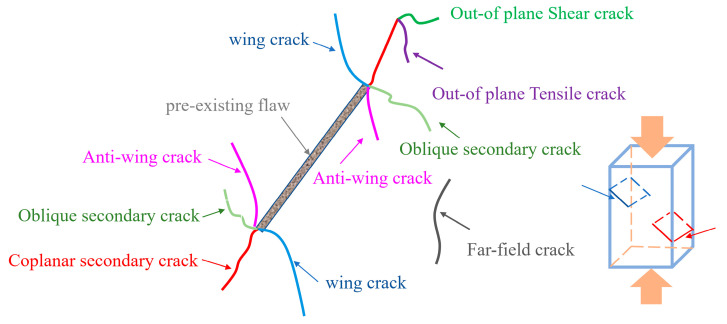
Main crack types under uniaxial compression.

Obtaining the whole process of strain field evolution through DIC technology is an effective method to analyze crack propagation and failure mode [30,31]. Figure 15 shows the main crack types of jointed rock under uniaxial compression.

#### 3.3.1. Specimens with Different Rock Bridge Lengths, L

**Figure 16:**
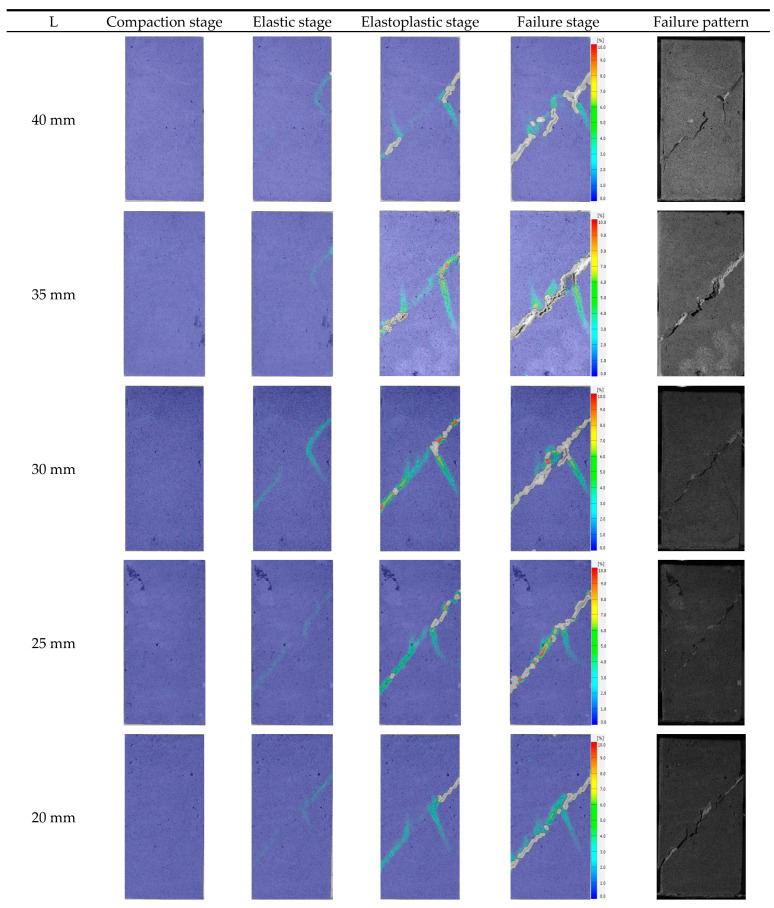
Maximum principal strain field of specimens with different L.

Figure 16 shows the principal strain field evolution of specimens with different L. The principal strain fields of different specimens show similarities in the compaction stage, elastic stage, and elastic-plastic stage: (1) Compaction stage: the internal stress is small, the failure mainly consists of compaction of original defects and pores. The displacement at each speckle is very small, and the principal strain field is basically unchanged; (2) Elastic stage: obvious strain occurs at the prefabricated joints. Occasionally, the wing cracks at the tips of prefabricated joints will propagate, and the deformation is generally in an elastic stage; (3) Elastoplastic stage: large strain occurs at the prefabricated joints, accompanied by surface spalling and falling off, and the gray cement mortar inside is visible. The wing cracks at the right joint tips propagate downward obviously. Sometimes, the wing crack propagates upward from the left joint tip, and the strain value is relatively small. In addition, although the failure mode of specimens is shearing failure through the rock bridge along the direction of prefabricated joints, specimens with different L show different failure mechanisms in the failure stage. When L is relatively large (L = 40 mm, 35 mm), the failure plane is composed of the coplanar shear crack at the joints’ tips and the out-of-plane shear crack, which eventually leads to the coalescence of the rock bridge and the formation of shear failure. The main cracks include one shear failure plane and two wing cracks, and the coalescence degree is higher when L = 35 mm. When L is small (L= 30 mm, 25 mm, 20 mm), coplanar shear cracks initiated from the joints’ tips directly coalesce with each other, forming the final failure plane; the main cracks include one shear failure plane and one wing crack. With the decrease of L, the formation of the failure plane changes from the coalescence of coplanar shear cracks and out-of-plane shear cracks to the direct coalescence of coplanar shear cracks. In practical engineering, the length of the rock bridge should be limited to a certain range.

#### 3.3.2. Specimens with Different Rock Bridge Angles, α

**Figure 17:**
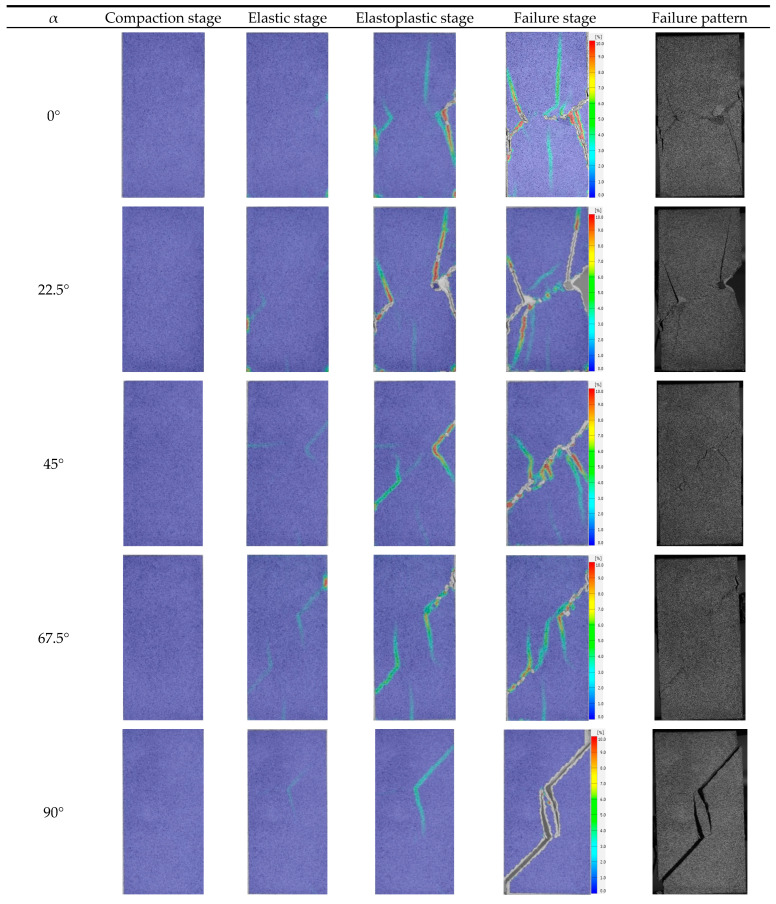
Maximum principal strain field of specimens with different α.

Figure 17 shows the principal strain field of specimens with different α. Due to the large differences in the evolution process of the displacement field under different α, this paper describes them separately.

When α = 0°, the evolution process of the principal strain field is as follows: (1) Compaction stage: the failure is mainly dominated by the compaction of the primary defect, the surface displacement is very small, and the principal strain field has no obvious change; (2) Elastic stage: elastic deformation mainly occurs inside the specimen, and strain bands only appear at the prefabricated joint; (3) Elastoplastic stage: wing cracks develop and expand at the tips of prefabricated joints, the right wing cracks almost expand to the edge of the specimens, and a crack initiates above the rock bridge; (4) Failure stage: there is one macrocrack in the rock bridge area, and wing cracks continue to expand to the sides of the specimen; surface spalling also appears in this stage. The crack above the rock bridge continues to expand, and a crack initiates below the rock bridge. The stress decreases slightly at this stage, and with the continuous increase of displacement, the crack above the rock bridge expands to the joint tip and the specimen fails immediately. It can be seen that the main failure plane consists of macrocracks, which coalesce at the rock bridge, wing cracks, and vertical cracks. The failure modes are mainly split along the loading direction, with shear failure along the rock bridge direction.

When α = 22.5°, the change of the principal strain field is divided into four stages: (1) Compaction stage: the primary defects are compacted, the surface displacement is very small, and the principal strain field shows no obvious change; (2) Elastic stage: the failure inside the specimen is mainly elastic deformation, and there are stain bands initiated only from the joint tips; (3) Elastoplastic stage: one wing crack is generated at the left tip of the prefabricated joint, and two wing cracks are initiated at the right tip. Some wing cracks have already coalesced; (4) Failure stage: the main macrocrack, which coalesces with the rock bridge, and the two wing cracks continue to develop and expand until the rock is destroyed. A new wing crack is initiated at the right tip, and four wing cracks are propagated on the left and right sides of the specimen. The continuous propagation and coalescence of the wing cracks are the main reason for the fluctuation near the peak stress and the step rise of cumulative AE count-time curve. After the final failure, it can be seen that the failure plane occurs when the macroscopic crack coalesces with the rock bridge and wing cracks develop at the tips of the prefabricated joints, and the failure mode is mainly shear failure along the rock bridge direction.

When α = 45°, the variation of the principal strain field is also divided into four stages. The strain field in the compaction stage and elastic stage is almost same as other specimens. In the elastoplastic stage, wing cracks initiate from the joint tips, and there is also a shear strain band located in the rock bridge area. After entering the failure stage, a crack coalescing with the rock bridge is generated, wing cracks propagate to the sides of the specimen, and surface spalling occurs in the rock bridge area due to shearing. After the failure, it can be seen that the main cracks are macroscopic cracks, which coalesce with the rock bridge, and wing cracks that develop at the joint tips; the failure mode is mainly shear failure along the rock bridge direction.

For specimens with α = 67.5°, the strain field in the first two stages is almost same as the specimen with a joint angle of 22.5–45°, but the crack propagation angle is a little different in this stage. In the elastoplastic stage, two wing cracks initiate from the joint tips, and there is no significant strain variation in the rock bridge area. In the failure stage, the rock bridge coalesces, wing cracks do not propagate, and surface spalling also occurs. The main cracks are macroscopic cracks, which coalesce with the rock bridge, and wing cracks that initiate at the joint tips; the failure plane consists of an out-of-plane tensile crack and two coplanar shear cracks initiated from the joint tips. According to Wen et al. [32] and Zhang et al. [15], for rock with two edge joints (joint angle of 45° and ligament angle of 60°), the dominant factor for the coalescence mode is out-of-plane shear cracking. It can be inferred that the critical angle for the transition from the out-of-plane shear crack dominant mode to the out-of-plane tensile crack dominant mode is between 60° and 67.5°.

When the joint angle equals 90°, the strain field begins to change at the right prefabricated joint. This strain band continues to propagate during the whole elastoplastic stage. There is no strain band generated in rock bridge area in the first three stages. In the failure stage, wing cracks initiated from the joint tips propagate along the loading direction, and the specimen finally fails when wing cracks coalesce with the coplanar shear cracks initiated from the joint tips.

Based on the above analysis, it can be seen that when α is small, shear failure occurs, accompanied with multiple tensile cracks. With the increase of α, the number of tensile cracks generated during the whole failure process decreases, and the coalescence degree of the rock bridge increases. From 45° to 90°, the dominant crack changes from out-of-plane shear cracking to out-of-plane tensile cracking and then to wing cracks. For rock with internal joints, as the ligament angle increases, the coalescence mode will directly transmit from the out-of-plane shear crack dominant mode to the wing crack dominant mode, lacking an out-of-plane tensile crack dominant mode [33]. The transition of the coalescence mode for rock with edge joints is more complex. Tang et al. [34] studied the influence of the ligament angle on the coalescence mode of rock with edge joints through changing joint inclination angle while maintaining the position of the joint outer tip. The variation of the coalescence mode is consistent with the finding in this study. It can be seen that the coalescence mode between joints is mainly influenced by the geometric parameters of the rock bridge, and the influence of joint geometric parameters is relatively weak. As wing cracks are easier to initiate than out-of-plane cracks, the rock bridge area of specimens with higher α is easier to coalesce, and the proportion of shear microcracks is also highest for specimens with α = 90°. The increase of α makes the specimen a more vulnerable structure under loading. In practical engineering, rock mass with high α should be avoided as much as possible.

## 4. Conclusions

To provide a theoretical basis for predicting and preventing the disaster of rock mass in deep foundation pits, this study takes the common intermittent jointed rock mass in deep foundation pit excavation as the research object, carries out a series of laboratory tests under different rock bridge conditions, and uses DIC acoustic emission technology for analysis. The conclusions are listed as follows:

(1) The UCS shows a linear increase with L. This is attributed to the enhanced control effect of increasing L on specimens with intermittent joints. Conversely, the uniaxial compressive strength exhibits a nonlinear decline with α. As α increases, the specimen becomes more susceptible to damage, and when α > 45°, the UCS experiences a more pronounced decline. The length and angle of the rock bridge are the key points of geological investigation of the foundation pit rock mass.
(2) The AE count and cumulative AE count increase with applied load. The expansion and coalescence of wing cracks and macroscopic cracks contribute to the surge in AE count and cumulative AE count. Analysis of RA-AF values indicates that the specimens are primarily characterized by shear damage. The smaller the L, and the larger the α, the higher the proportion of shear damage, with α exerting a greater influence on the form of damage. The number of shear microcracks surpasses that of tensile microcracks, and the prevalence of shear microcracks intensifies as loading progresses, reaching a peak at the conclusion of loading. The higher the proportion of shear cracks, the greater the risk of rock mass disaster in foundation pit excavation.
(3) The length (L) and angle (α) of the rock bridge significantly influence the failure mode. As L decreases, the failure plane that crosses rock bridge area transforms from the coalescence of two coplanar shear cracks and one out-of-plane shear crack to direct coalescence of coplanar shear cracks, resulting in a reduced number of tensile cracks required for failure. In cases where α is small, the specimen undergoes shear failure only in the presence of multiple and highly developed tensile cracks. Conversely, as α increases, the number of tensile cracks necessary for specimen failure decreases, and the coalescence degree of the rock bridge is higher. Consequently, it is advisable to use measures to limit the length and angle of rock bridges during pit excavation and support.

This manuscript only focused on the relationship between the coalescence mode between joints and rock bridge geometric parameters under uniaxial loading. However, the stress situation of rock is often more complex and may involve biaxial compression or compressive-shear loading. It is of significance to study the coalescence mechanism of a rock bridge under complex loading conditions. In addition, analyzing the failure behavior of rock through the AE RMS method is also worth exploring in the future.

## Data Availability

The data presented in this study are available on request from the corresponding author.
